# Inhibitory Effects of the Ruthenium Complex KP1019 in Models of Mammary Cancer Cell Migration and Invasion

**DOI:** 10.1155/2009/681270

**Published:** 2009-09-17

**Authors:** A. Bergamo, A. Masi, M. A. Jakupec, B. K. Keppler, G. Sava

**Affiliations:** ^1^Callerio Foundation Onlus, Via A. Fleming 22-31, 34127 Trieste, Italy; ^2^Institute of Inorganic Chemistry, University of Vienna, Waehringer Strasse 42, 1090 Vienna, Austria; ^3^Department of Life Sciences, University of Trieste, Via L. Giorgieri 7, 34127 Trieste, Italy

## Abstract

The effects of indazolium *trans*-[tetrachlorobis(1H-indazole)ruthenate(III)] (KP1019, or FFC14A), the second ruthenium compound that entered clinical trials, in an in vitro model of tumour invasion and metastasis show that the antitumour effects of this compound might include also the modulation of cell behaviour although its cytotoxicity appears to be predominant over these effects. The comparison with its imidazole analogue KP418 shows however its superiority, being able to control in vitro cell growth and in some instances also in vivo tumour development. These results suggest that the activity of KP1019 is predominantly due to direct cytotoxic effects for tumour cells, evident also in vivo on primary tumour growth and that the effects on modulation of the biological behaviour of the cancer cell can be present but might have only a partial role.

## 1. Introduction

The research on anticancer innovative drugs based on the ruthenium metal received a strong impulse from the extensive knowledge of the coordination and redox properties of metal ions. The knowledge of the role of the “activation by reduction” hypothesis [1, 2], suggesting the reduced Ruthenium(II)species as the final reactive drug, prompted the development of Ruthenium(III) compounds with the aim of getting prodrugs activated selectively in the hypoxic environment of solid tumours. The study of complexes with a redox potential accessible to the biological redox environment has led to the identification of the antitumour properties of several complexes among which the series of so-called “ruthenium bis-heterocycle” complexes characterised by heterocycle ligands placed in the axial position have been tested in vitro and in vivo. One of them, namely, indazolium *trans*-[tetrachlorobis(1H-indazole)ruthenate(III)] (KP1019 or FFC14A) is receiving attention for its activity against various tumor types in vivo as indicated by stable disease seen in heavily pretreated cancer patients in a phase I trial [3]. 

**Figure 1:**
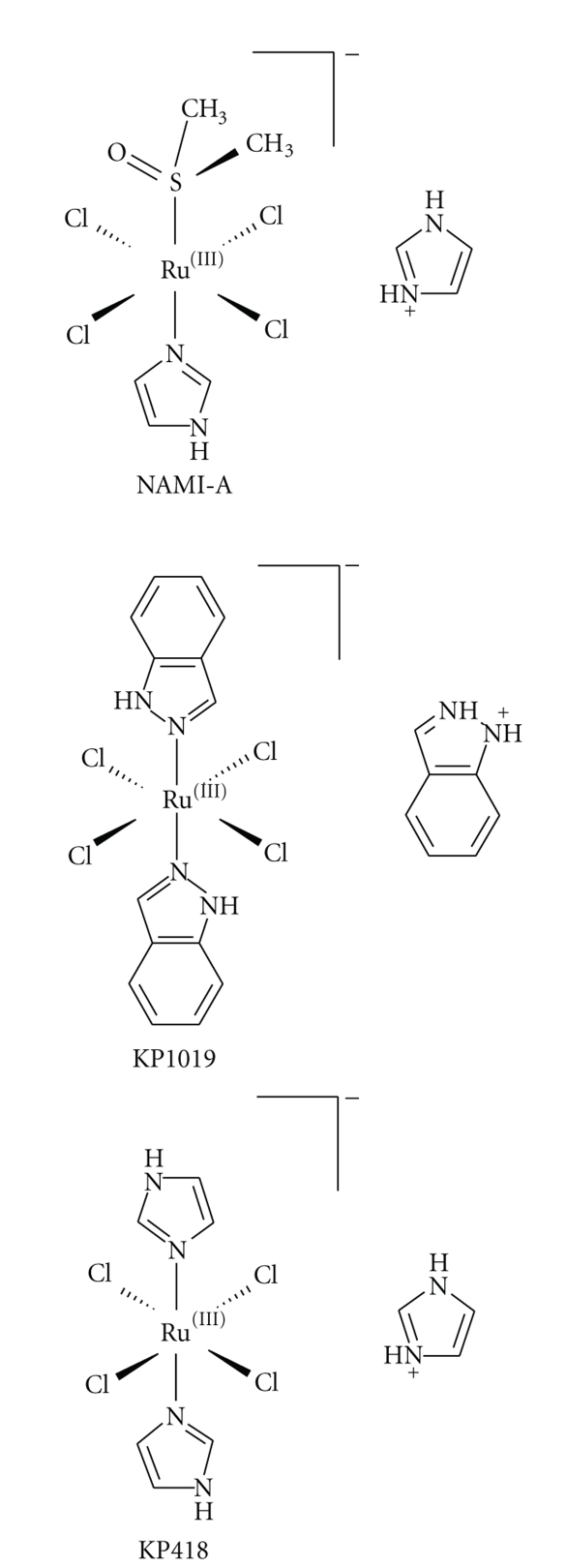
Chemical structures of NAMI-A, KP1019, and KP418.

The aim of this study is to evaluate the effects of KP1019, in comparison to its bis-imidazole analogue KP418 (Figure 1), in an in vitro model of tumour invasion and metastasis. Although isostructural with KP1019, KP418 showed a slower reduction rate [4], but higher toxicity in therapeutically active doses in vivo in a model of chemically induced autochthonous colorectal cancer [5] and very low cytotoxicity in human cancer cell lines [6]. Effects of KP1019 (but not KP418) on adhesion properties of cancer cells were first suggested by the simple observation that human SW480 colon carcinoma cells resist harvesting by trypsinisation upon treatment with KP1019 in concentrations in the 10^−4^ M range for as short as 30 minutes [6]. Microscopic examination revealed structures tentatively considered as stress fibres (B. Marian, personal communication). Although not examined in detail, these observations were reminiscent of the strong actin-dependent adhesion of tumour cells induced by short-term exposure to the investigational antimetastatic ruthenium-based drug NAMI-A [7], raising the question whether KP1019 is capable of affecting cellular properties related to the processes of tumour invasion and metastasis.

The metastatic progression is mimicked in vitro by opportune experiments to study cell detachment from the primary tumour, extracellular matrix degradation, cell ability to migrate in response to chemical or contact stimuli, invasion, and readherence to a substrate, with the use of cell lines of the mammary gland with different degree of aggressiveness: MDA-MB-231, a highly invasive breast cancer cell line, MCF-7, a tumorigenic but not invasive cell line, and HBL-100, a non-tumorigenic cell line of the mammary epithelium. The in vitro study is compared with the antitumour and antimetastatic effects of the same compounds in vivo in the mouse model of MCa mammary carcinoma.

## 2. Materials and Methods

### 2.1. Drugs and Reagents

KP418 and KP1019 were prepared according to the published procedures [8, 9]. All reagents were purchased from Sigma-Aldrich (St. Louis, Mo, USA) unless otherwise indicated.

### 2.2. Tumor Cell Lines for In Vitro Tests

The MDA-MB-231 human highly invasive breast cancer cell line was kindly supplied by Dr. P. Spessotto (Cro, Aviano, Italy), and maintained in Dulbecco's modified Eagle's medium (EuroClone, Devon, UK) supplemented with 10% FBS (Gibco, Invitrogen, Paisley, Scotland, UK), 2 mM L-glutamine (EuroClone, Devon, UK), 1% non-essential aminoacids, and 100 IU/mL penicillin and 100 *μ*g/mL streptomycin (EuroClone, Devon, UK).

The MCF-7 human breast cancer cell line was obtained from the American Type Culture Collection (Manassas, VA; catalogue number HTB-22) and maintained in Dulbecco's modified Eagle's medium/F12 medium 1 : 1 v/v (EuroClone, Devon, UK) supplemented with 10% FBS, 2 mM L-glutamine, and 100 IU/mL penicillin as well as 100 *μ*g/mL streptomycin.

The HBL-100 human non-tumorigenic epithelial cell line was kindly supplied by Dr. G. Decorti (Department of Biomedical Sciences, University of Trieste, Italy), and maintained in McCoy's 5A medium supplemented with 10% FBS, 2 mM L-glutamine, and 100 IU/mL penicillin and 100 *μ*g/mL streptomycin.

All cell lines were kept in a CO_2_ incubator with 5% CO_2_ and 100% relative humidity at 37°C. Cells from a confluent monolayer were removed from flasks by a trypsin-EDTA solution. Cell viability was determined by the trypan blue dye exclusion test [10]. For experimental purposes cells were sown in multiwell culture clusters.

### 2.3. Resistance to Detachment Assay

The cells ability to resist detachment after treatment with KP418 and KP1019 was measured by the following. 96 well plastic plates (Corning Costar, Milano, Italy) were coated with the following substrates: 10 *μ*g/mL poly-L-lysine, 20 *μ*g/mL fibronectin from human plasma, and 20 *μ*g/mL collagen IV from human placenta, and left in a humidified cell-culture chamber at 37°C for 4 hours. Before cell seeding, plates were washed with CMF-DPBS (Calcium and Magnesium Free Dulbecco's Phosphate Buffered Saline), then 6 · 10^3^cells in 0.2 mL complete medium were sown in each well. After 2 days at 37°C, complete medium was replaced with serum-starved medium, containing 0.1% w/v BSA. After 24 hours the medium was removed and the plates washed with CMF-DPBS, before the treatment with KP418 and KP1019 10^−4^ M, dissolved in DPBS (Dulbecco's Phosphate Buffered Saline), was added to the wells and incubated for 1 hour. At the end of the treatment the KP418- and KP1019- containing solutions were removed, the plates were washed twice with CMF-DPBS, and trypsin solution, 0.008% w/v, was added to each well. Plates were kept in agitation for 30 minutes at room temperature then trypsin solution was removed and wells washed with CMF-DPBS. Cells that still adherent to the plates were detected by the sulforhodamine B (SRB) test. Resistance to detachment is expressed as arbitrary units, calculated by dividing the mean absorbance of treated cells by the mean absorbance of control cells.

### 2.4. Re-adhesion Assay

The effect of the ability of the cells to readhere after KP418 and KP1019 treatment was studied in cells maintained for 24 hours in serum-starved medium, and then treated for 1 hour with KP418 and KP1019 10^−4^ M in DPBS. At the end of the treatment cells were removed from flasks by a trypsin-EDTA solution, collected by centrifugation, resuspended in serum-starved medium supplemented with 0.1% w/v BSA and kept for 30 minutes at room temperature to allow surface receptor reconstitution. The cells were then seeded at a density of 1·10^4^ cells in 0.1 mL/well on 96 well plastic plates previously coated as described above with poly-L-lysine, fibronectin, collagen IV or 20 *μ*g/mL Matrigel (BD, Biosciences, San Josè, Calif, USA). Cells were left to adhere for 30 and 60 minutes at 37°C with 5% CO_2_ and 100% relative humidity, then the medium containing the non-adherent cells was removed and wells were gently washed with CMF-DPBS. Cells that in 30 or 60 minutes have adhered to the substrates were detected by the sulforhodamine B (SRB) test.

### 2.5. Sulforhodamine B Assay

Adherent cells were detected with the SRB test described by Skehan et al., [11]. Briefly, adherent cells were fixed with 10% v/v cold trichloroacetic acid (TCA) at 4°C for 1 hour. After fixation TCA was discarded and wells washed five times with distilled water and air-dried. SRB solution (0.4% w/v, in 1% acetic acid) was added to the wells and plates were kept for 30 minutes at room temperature. Unbound SRB was removed by washing three times with 1% acetic acid. Plates were air dried, then bound stain was dissolved with un-buffered 10 mM Tris base (tris-hydroxymethyl-aminomethane) at pH 10.5 and the optical density was read at 570 nm with an automatic computerised spectrophotometer (SpectraCount; Packard, Meriden, Conn, USA).

### 2.6. Migration Assays

Migratory ability resulting from a haptotactic or a chemotactic stimulus was measured in Transwell cell culture chambers (Costar, Milano, Italy). In the haptotaxis assay the lower surface of the a polyvinylpyrrolidone-free polycarbonate filter (8-*μ*m pore size) was coated with 10 *μ*g/mL fibronectin and left in a humidified cell culture chamber at 37°C for 2 hours, then washed with CMF-DPBS before cell seeding. In the chemotaxis assay inserts were used without coating. Cells were treated for 1 hour with KP418 and KP1019 10^−4^ M and with KP1019# (# is equal to 10^−6^ M for MDA-MB-231 and MCF-7 and to 10^−5^ M for HBL-100) in DPBS. After treatment, cells were removed with a trypsin-EDTA solution, collected by centrifugation, resuspended in serum-starved medium supplemented with 0.1% w/v BSA, and 1·10^5^ cells in 0.2 mL were sown in the upper compartment of each chamber. The lower compartment was filled with serum-starved medium supplemented with 0.1% w/v BSA, and with complete medium for the haptotaxis and the chemotaxis assay, respectively.

Cells were left to migrate for 24 hours, then the cells on the upper surface of the filters were removed with a cotton swab and migrating cells, present in the lower surface, were detected by the crystal violet assay.

### 2.7. Invasion Assay

Invasive ability was measured in a Transwell cell culture chamber according to the method of Albini et al. [12]. Briefly, the upper surface of the polycarbonate filter (8 *μ*m pore size) of Transwell cell culture chambers was coated with 50 *μ*L of a 600 *μ*g/mL Matrigel solution and air dried overnight at room temperature. The filters were reconstituted with DMEM medium for 90 minutes under gently shaking immediately before use. Cells were treated as described for the migration assays and 0.5·10^5^ cells in 0.2 mL were sown in each chamber. Cells were left invade for 96 hours, then the cells on the upper surface of the filters were removed with a cotton swab and invading cells, present in the lower surface, were detected by the crystal violet assay.

### 2.8. Crystal Violet Assay

The crystal violet assay was performed according to the method described by Kueng et al., [13]. Briefly, the cells present on the lower surface of the filter were fixed with a 1.1% w/v glutaraldehyde solution for 15 minutes. After fixation the wells were washed three times with distilled water and air dried. Cells were stained for 20 minutes with 0.1% w/v crystal violet prepared in 200 mM boric acid, pH 9.0, then washed three times with distilled water and air-dried prior to dissolve the dye with 10% acetic acid solution. The optical density was read at 590 nm with an automatic computerised spectrophotometer (SpectraCount; Packard, Meriden, Conn, USA).

### 2.9. Cell Viability

To evaluate if treatment with KP418 and KP1019, in the experimental conditions adopted, can affect cell viability, cells were treated as described above for migration tests, except they were seeded on 96 well plates. Briefly, cells treated in culture flasks for 1 hour with 10^−4^ M KP418 or with 10^−6^, 10^−5^, and 10^−4^ M KP1019 were detached and sown on 96-well plates and, after additional 24 hours incubation in culture medium, cell viability was detected by the MTT viability test [14]. Briefly, a solution of MTT [3-(4,5-dimethylthiazol-2-yl)-2,5-diphenyltetrazolium bromide] dissolved in CMF-DPBS (5 mg/mL) was added to each well (10 *μ*L per 100 *μ*L of medium) and the plates were incubated at 37°C with 5% CO_2_ and 100% relative humidity for 4 hours. After this time, the medium was discarded and 200 *μ*L of DMSO were added to each well to dissolve the formazan crystals. The optical density was measured at 570 nm with an automatic computerised spectrophotometer (SpectraCount; Packard, Meriden, Conn, USA).

### 2.10. Zymography

To visualise the direct effect of KP418 and KP1019 on the activity and/or production of MMP-2 and MMP-9 enzymes, sodium dodecyl sulphate (SDS) polyacrylamide gel electrophoresis (PAGE) zymography was carried out with conditioned medium of MDA-MB-231 and HBL-100 cells. Cells at 70% confluence were incubated for 24 hours in serum-starved medium containing 0.1% BSA, before being treated with KP418 and KP1019 10^−4^ M for 1 hour. At the end of the treatment, the KP418 and KP1019 solutions were discarded and complete serum-free medium containing 0.1% w/v BSA was added for a further 24 hours, when culture media were collected, centrifuged to remove cellular debris, then concentrated approximately 15 times using Amicon Ultra-15 30,000 nominal molecular weight limit centrifugal filter devices (Millipore Corporation, Bedford, Mass, USA). The conditioned media obtained were stored at −80°C until use. Equal amounts of proteins, as determined by the Bradford method [15], for each sample were eluted with Laemmli non-reducing sample buffer and analysed by SDS-PAGE on a 7% polyacrylamide gel containing 0.1% w/v gelatine. At the end of electrophoresis in a dual-laboratory system (Protean II, Bio-Rad Laboratories, Hercules, CA, USA), the gels were washed two times for 30 minutes at 4°C in 2.5% Triton X-100 to remove SDS. After additional washing in water (three times for 5 minutes), the gels were incubated at 37°C overnight in collagenase buffer (200 mM NaCl, 50 mM tris(hydroxymethyl)aminomethane, 5 mM CaCl_2_, adjusted to pH 7.4) to reactivate enzyme activity. The gels were then stained with 0.5% Coomassie brilliant blue. The gelatinolytic regions were observed as white bands against a blue background. Quantitative evaluation of the band intensity, on the basis of grey levels, was performed using Image Master 2D version 4.01 and Magic Scan 32 version 4.3 software.

### 2.11. In Vivo Tests

The in vivo experiments were carried out with the murine mammary carcinoma (MCa), originally obtained from the Department of Biology, Rudjer Boskovich Institute (Zagreb, Croatia), grown in CBA female mice, obtained from a local breeding colony grown according to the standard procedures for inbred strains [16]. The tumour line was locally maintained by serial biweekly passages of 10^6^ viable tumour cells, of a cell suspension prepared from mincing (with scissors) the primary tumour masses obtained from donors similarly implanted 2 weeks before. The minced tissue was filtered through a double layer of sterile gauze, centrifuged at 250 xg for 10 minutes, and resuspended in an equal volume of CMF-DPBS; viable cells were counted by the trypan blue exclusion test. 10^6^ viable tumour cells were injected I.M. into the left hind calf of experimental groups. KP1019 was administrated as 10% DMSO solution in sterile saline (0.9% NaCl) and given to mice by I.P. administrations at two dose levels of 40 mg/kg/day from day 6 to day 11 after tumour implant, and 80 mg/kg/day on days 7, 9, and 11 after tumour implant.

Primary tumour growth was determined by calliper measurements, by measuring two orthogonal axes, and the tumour volume was calculated with the formula: (Π/6) *xa* ^2^ *xb*, where *a* is the shorter axis and *b* the longer axis, assuming tumour density equal to 1 g/mL. The evaluation of the number and weight of lung metastases was performed by examining the surface of the lungs immediately after sacrificing the animals by cervical dislocation. Lungs were dissected into five lobes, washed with CMF-DPBS, and examined under a low power microscope equipped with a calibrated grid. The weight of each metastasis was calculated by applying the same formula used for primary tumours and the sum of each individual weight gave the total weight of metastatic tumour per animal.

### 2.12. Animal Studies

Animal studies were carried out according to guidelines enforced in Italy (DDL 116 of 21/2/1992 and subsequent addenda) and incompliance with the Guide for the Care and Use of Laboratory Animals(National Academy Press, Washington*, *D.C. CSSG).

### 2.13. Statistical Analysis

Results were subjected to computer-assisted statistical analysis using the One-Way Analysis of Variance ANOVA, and the Tukey-Kramer post-test. Differences of *P* < .05 were considered to be significantly different from the controls.

## 3. Results

### 3.1. Resistance to Detachment

**Figure 2:**
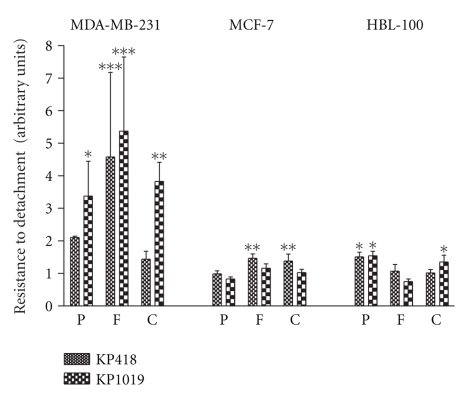
Effect of KP418 and KP1019 on resistance to detachment. MDA-MB-231, MCF-7, and HBL-100 cells seeded on 96-well plastic plates previously coated with poly-L-lysine, fibronectin and collagen IV, exposed for 1 hour to KP418 and KP1019 10^−4^ M and then to a diluted trypsin solution for 30 minutes, before detecting cells still attached to the growth substrate by the SRB test. Arbitrary units are calculated from the mean ± S.D. of two experiments, each performed in quadruplicate. *, *P* < .05; **, *P* < .01; ***, *P* < .001 versus controls, ANOVA, and Tukey-Kramer post test.

The resistance to detachment is an index of the propensity of tumour cells to detach from the primary site of growth with the aim to disseminate. This ability was studied by seeding cells on components of the extra cellular matrix (ECM) such as fibronectin (F) and collagen IV (C) and, for comparison, on poly-L-lysine (P) a substrate on which cells simply adhere by electrostatic interactions (Figure 2). KP1019 increased the resistance to detachment of the MDA-MB-231 cells much better than that of the low invasive MCF-7 cells or the non-tumorigenic HBL-100 cells, independently of the substrate on which cells are grown. KP418 showed a similar behaviour and globally its effects were lower than those of KP1019.

### 3.2. Re-adhesion after Treatment

**Figure 3:**
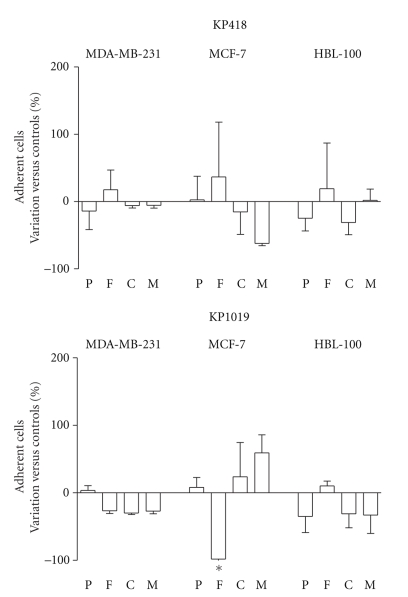
Effect on ability of cells to readhere after KP418 and KP1019 treatment. MDA-MB-231, MCF-7, and HBL-100 cells were treated for 1 hour with KP418 and KP1019 10^−4^ M, then the cells were removed from the flasks, collected, resuspended and seeded on 96-well plastic plates previously coated with poly-L-lysine, fibronectin, collagen IV and Matrigel. After 30 and 60 minutes of incubation cells that adhered to the substrates were detected by the SRB test. Data are the percent of variation versus controls calculated from the mean ± S.D. of two experiments, each performed in triplicate. *, *P* < .05 versus controls, ANOVA, and Tukey-Kramer post test.

The propensity to readhere to fibronectin (F), collagen IV (C), and Matrigel (M), in comparison to poly-L-lysine (P), of MDA-MB-231, MCF-7, and HBL-100 cells, following 1 hour challenge with 10^−4^ M KP1019 and KP418 was studied exposing cells to the compounds while they were adherent to the growth substrate (Figure 3). The two metal compounds show no significant modifications of the cell ability to readhere after treatment with the exception of KP1019 that significantly reduces the attachment of MCF-7 to fibronectin.

### 3.3. Effects on Migration and Invasion

**Figure 4:**
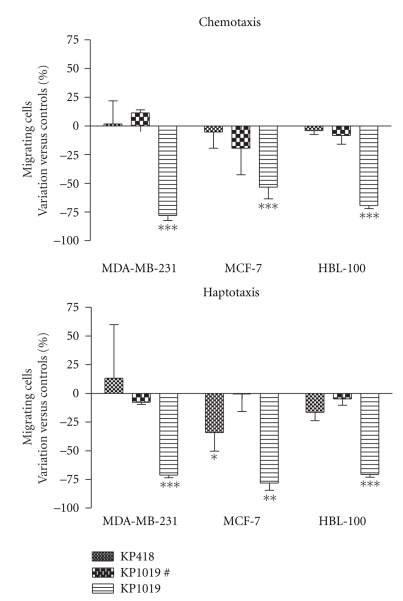
Effect of KP418 and KP1019 on migration of cells through polycarbonate filters. MDA-MB-231, MCF-7, and HBL-100 cells were treated for 1 hour with KP418, KP1019 10^−4^ M, and KP1019# (# is equal to 10^−6^ M for MDA-MB-231 and MCF-7 and to 10^−5^ M for HBL-100), then the cells were removed from the flasks, collected, resuspended, and seeded on the inserts of Transwell cell culture chambers. Data represent cells that after 24 hours have migrated and are present on the lower surface of the filter. Data are the percent of variation versus controls calculated from the mean ± S.D. of two experiments each performed in triplicate, *: *P* < .05; **: *P* < .01; ***: *P* < .001 versus controls, ANOVA, and Tukey-Kramer post test.

The effects of KP1019 and KP418 on cell migration were determined with properly adapted Transwell chambers, where the cells were subjected to a chemical (chemotaxis) or a contact (haptotaxis) stimulus to promote cell movement (Figure 4). Treatment with 10^−4^ M KP1019 for 1 hour caused a statistically significant and pronounced reduction of cell migration, independently of the stimulus being applied and of the cell line being used. 10^−4^ M KP418 is almost completely devoid of effects in these tests with the exception of a mild reduction of cell response to haptotaxis of MCF-7 cells.

**Figure 5:**
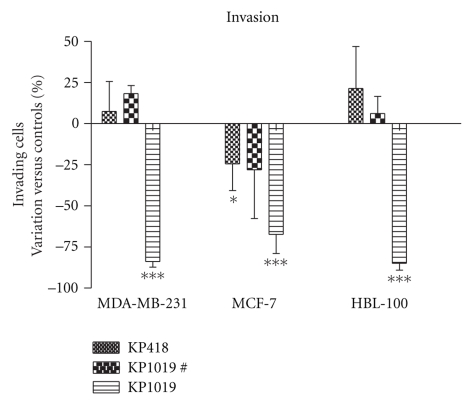
Effect of KP418 and KP1019 on invasion of cells through Matrigel. MDA-MB-231, MCF-7, and HBL-100 cells were treated for 1 hour with KP418, KP1019 10^−4^ M, and KP1019# (# is equal to 10^−6^ M for MDA-MB-231 and MCF-7 and to 10^−5^ M for HBL-100), then the cells were removed from the flasks, collected, resuspended and seeded on inserts. Data represent cells that after 96 hours have invaded and are present on the lower surface of the filter. Data are the percent of variation versus controls calculated from the mean ± S.D. of two experiments, each performed in triplicate, *: *P* < .05: ***: *P* < .001 versus controls, ANOVA, and Tukey-Kramer post test.

The invasion ability of the same cells, studied on Transwell chambers coated with a 3D matrix, (Figure 5) was similarly affected by 10^−4^ M KP1019 while 10^−4^ M KP418 again reduced the invasion of only MCF-7 cells.

### 3.4. Effect on Cell Viability

The study of the effects of KP1019 and KP418 on cell viability was done in the same condition at which the two ruthenium complexes were tested on cell migration, that is, 1 hour at 10^−4^ M, or lower doses in the case it is found to significantly reduce cell growth as compared to untreated controls, with analysis after additional 24 hours in culture medium. Data reported in Table 1 show KP418 to cause a very mild reduction of cell growth of the invasive MDA-MB-231 cells with non appreciable effects on the less malignant MCF-7 cells or the non-tumorigenic HBL-100 cells. KP1019, in the same experimental conditions, was much more cytotoxic, maintaining a certain degree of cytotoxicity also at the lower dosage of 10^−5^ M.

### 3.5. Effect on MMPs Production and Activity

**Figure 6:**
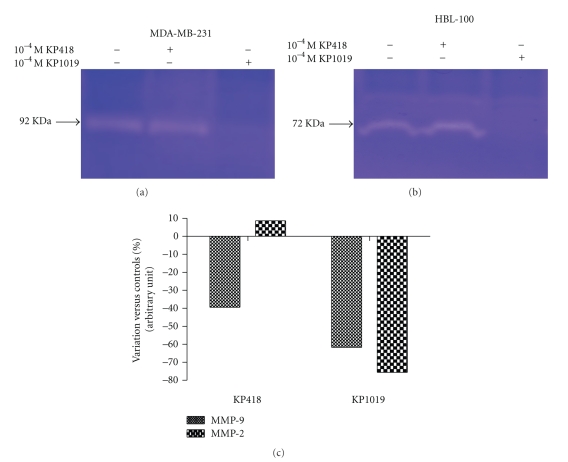
Effect of KP418 and KP1019 on MMPs production and/or activity. MDA-MB-231, and HBL-100 cells were treated for 1 hour with KP418 and KP1019 10^−4^ M, then incubated for additional 24 hours in serum-starved complete medium containing 0.1% BSA. Supernatants containing MMPs were collected and concentrated and equal protein amounts (100 *μ*g) subjected to SDS-PAGE. Gelatine digestion by proteases is detected as white bands against a blue background (a, b). Band digestion is quantified by using Image Master 2D version 4.01 and Magic Scan 32 version 4.3 software (c).

The effects of the two metal compounds on the matrix metallo proteinases (MMP-2 and MMP-9) production and/or activity were studied with the gelatine zymography test. MDA-MB-231 cells produce the 92 KDa MMP-9 in appreciable amounts whereas HBL-100 cells prevalently produce the 72 KDa MMP-2. KP1019 reduced the production/activity of both MMP-2 and MMP-9 by approximately 65–75% of controls, whereas KP418 reduced MMP-9 activity by about 40% of controls (Figure 6).

### 3.6. Effect on Lung Metastases In Vivo

The effects of KP1019 treatment on primary tumour growth and lung metastasis formation were studied in the model of MCa mammary carcinoma, a murine transplantable tumour that spontaneously metastasise to the lungs (Table 2). The daily administration of 40 mg/kg/day from day 6 to day 11, or 80 mg/kg/day on days 7, 9, and 11 after tumour implantation caused a comparable and statistically significant reduction of the growth of the intramuscular tumour more pronounced at the end of the treatment schedule than a week later. None of the treatments reduced the development of lung metastatases in this tumour model.

## 4. Discussion

KP418 is mostly known for its capacity to reduce the growth of an autochthonous colorectal cancer induced by carcinogens in the rat [5]. In spite of its similarities with NAMI-A (they differ only in a ligand, imidazole in KP418 and dimethylsulfoxide in NAMI-A), KP418 shows a completely different pharmacological profile. Even though the biodistribution patterns of KP418 and NAMI-A are virtually identical in the Lewis lung carcinoma model [17], NAMI-A has no effect on the primary tumor but a significant effect on the metastases, while KP418 has a mild cytotoxic effect on the primary tumour but no significant effect on the growth of distant metastases. These differences might be attributed to the solution chemistry characteristics of these compounds, with NAMI-A being much more sensitive to reduction than KP418 [18].

From this point of view, KP1019 might be expected to combine the activities of both KP418 and NAMI-A, as it shares the cytotoxic effects on the primary tumour with the former and the redox sensitivity with the latter compound, as a consequence of the replacement of the two *trans* imidazoles with two indazole moieties [4]. In comparison with KP418, KP1019 had already proved to be at least as effective on primary colorectal tumours in the rat, but with the advantage of being nearly completely devoid of toxic side effects at the active doses used [5]. Moreover, the ability of KP1019 to induce enhanced adhesion of tumour cells (see Section 1) suggested a vague similarity with NAMI-A. In this study, we chose to investigate whether a component resembling the activity of NAMI-A, that is, reduction of tumour malignancy, is involved in the antitumour effects of KP1019 beside the previously described apoptotic mechanisms [6, 19].

Overall, the results of this study indicate that activity of KP1019 in murine mammary carcinoma in vivo is primarily not due to interference with the processes of tumour cell invasion and metastasis. Although inhibition of the primary tumour was demonstrated and inhibition of the increase of metastases weight suggested (without reaching statistical significance), the number of metastases was unaffected. We therefore conclude that any activity of KP1019 on metastases is due to direct cytotoxic effects, just as those on the primary tumor, rather than to the modulation of the biological behaviour of the cancer cell.

On the other hand, KP1019, unlike its isostructural analogue KP418, proved to be effective in a model mimicking cell invasion and metastasis in vitro. However, the data presented suggest that the strong inhibition of tumour cell invasion and the reduction of tumour cell migration upon chemotactic and haptotactic stimuli depend on the use of cytotoxic concentrations, but not on the malignancy of the tumour cell line, as the effects on the metastatic MDA-MB-231 cells equal those on the non-tumorigenic HBL-100 cells. Additionally, KP1019 clearly reduces the release of metalloproteases of the extracellular matrix (MMP-2 and MMP-9), enzymes often associated with unfavourable prognosis in many cancers including mammary tumours [20, 21].

These effects are indirectly dependent on the chemical consequences of the indazole ligands that replace the imidazoles of KP418, since KP418 is virtually devoid of effects in any of the tests performed. We have already noted that the redox potentials of KP1019 and NAMI-A are similar and biologically accessible, whereas KP418 is much less sensitive to reduction. Based on the observation that NAMI-A is active as an antimetastatic agent, while KP418 is not, it was hypothesized that sensitivity to biological reduction may play a role in antimetastatic agents. The data obtained with KP1019 suggest that this hypothesis may be incorrect. For NAMI-A, a drug for which the possibility to undergo reduction by biological reductants was repeatedly shown ([22] and references cited herein), this means that its selective effects on metastases might be due to other factors than to a simple reduction.

Finally, these data show that the challenge of the in vitro model simulating some of the most relevant steps of metastasis formation, with a cytotoxic ruthenium compound, such as KP1019, validates the claimed role of this model, highlighted with the lead NAMI-A (Callerio Foundation, confidential data), to identify compounds endowed of selective effects on metastases in the virtual absence of direct cell cytotoxicity, even when these effects are mild and less pronounced than those of NAMI-A, as reported with the organometallic compound RAPTA-T [23].

## Figures and Tables

**Table 1:** Effect of KP418 and KP1019 on cell viability.

	MDA-MB-231	MCF-7	HBL-100
Controls	2.284 ± 0.066	2.631 ± 0.055	2.308 ± 0.424
KP418 (10^−4^ M )	1.832 = 0.046*	2.763 ± 0.040	2.269 ± 0.159
	(79.9%)	(105%)	(97.0%)

	MDA-MB-231	MCF-7	HBL-100
Controls	1.482 ± 0.016	1.178 ± 0.073	0.999 ± 0.055
KP1019 (10^−6^ M)	1.435 ± 0.040	1.184 ± 0.051	1.076 ± 0.055
	(96.8%)	(101%)	(107%)
KP1019 (10^−5^ M)	1.361 ± 0.050***	1.04 ± 0.039*	0.901 ± 0.081
	(91.8%)	(88.3%)	(90.2%)
KP1019 (10^−4^ M)	0.847 ± 0.022***	0.862 ± 0.098***	0.328 ± 0.056***
	(57.2%)	(73.2%)	(32.9%)

MDA-MB-231, MCF-7, and HBL-100 cells were treated for 1 hour with KP418 10^−4^ M and KP1019 10^−6^ ÷ 10^−4^ M, then the cells were removed from the flasks, collected, resuspended in culture medium and seeded on 96 well plates. After 24 hours cell viability was determined by the MTT assay. Data are the mean optical density ± S.D. of two experiments each performed in quadruplicate. Data in parentheses represent the percentage of each treated group versus the relevant controls (T/C%), *: *P* < .05, ***: *P* < .001 versus controls, ANOVA, and Tukey-Kramer post test.

**Table 2:** Effect of KP1019 on lung metastases formation in mice with MCa mammary carcinoma.

(A)	Primary tumour weight (mg)		Lung metastases†	
	Day 13	Day 20	Number	Weight (mg)

Controls	1627 ± 286	3068 ± 616	29.2 ± 8.10	18.6 ± 12.1
KP1019 40 mg	1095 ± 592	2537 ± 563	31.9 ± 19.2	11.8 ± 11.8
	(67%)	(83%)	(109%)	(63%)

(B)	Primary tumour weight (mg)		Lung Metastases†	
	Day 13	Day 20	Number	Weight (mg)

Controls	1004 ± 174	2415 ± 439	17.5 ± 10.0	5.70 ± 6.90
KP1019 80 mg	527 ± 136.662***	1764 ± 370***	19.9 ± 23.2#	3.43 ± 5.83#
	(52%)	(73%)	(114%)	(60%)

Groups of 9 (experiment A) or 11 (experiment B) CBA mice, inoculated I.M. with 10^6^ MCa tumour cells on day 0 were treated I.P. with KP1019 at 40 mg/kg/day from day 6 to day 11 (A) and 80 mg/kg/day on days 7, 9, and 11 (B) after tumour implant. Data in parentheses are expressed as the percentage of the treated versus controls (T/C%), † Lung metastases were determined on day 20 after tumour implant, ***: *P* < .001 versus controls, ANOVA, and Tukey-Kramer post test, #: excluded the animals free of macroscopically detectable metastasis (2/11).
